# A novel device for resistance-free biomechanical testing of the metaphysis of long bones

**DOI:** 10.1186/1471-2474-15-245

**Published:** 2014-07-21

**Authors:** Gina Alicia Mackert, Christoph Hirche, Helmut Harhaus, Dimitra Kotsougiani, Bernd Hoener, Ulrich Kneser, Leila Harhaus

**Affiliations:** 1 Department for Hand-, Plastic- and Reconstructive Surgery – Burn Care Center – BG-Trauma Clinic Ludwigshafen, Department for Plastic Surgery of the University of Heidelberg, Ludwig-Guttmann-Str. 13, 67071 Ludwigshafen, Germany; 2 Technical and medical devices development and invention center, Remscheid, Germany; 3 Department of Social and Legal Sciences, SRH Hochschule Heidelberg, Ludwig-Guttmann-Str. 6, 69123 Heidelberg, Germany

**Keywords:** Biomechanics, Biomechanical testing, Bending and breaking test, Biomechanical devices, Metaphyseal bone, Osteoporosis

## Abstract

**Background:**

Biomechanical testing is an essential component of bone research. In order to test the metaphyseal region of long bones, a typical location for the nowadays increasing field of osteoporotic bone changes, three-point bending and breaking test devices are suitable and widely used. The aim of our study was to increase the effectiveness of this method by using a newly developed ball-mounted platform design. This new design eliminates the negative effects of friction, present in previous studies, caused by the lengthening of the distal tibia along its diaphyseal axis while sliding over the surface of a fixed aluminum block.

**Methods:**

70 tibiae of 35 twelve week old, female Sprague Dawley rats were separated into two groups for a metaphyseal bending/breaking test. Group 1 was made up of the rat’s right tibiae, Group 2 of the left tibiae. Group 1 was tested on a solid metal block according to previously established testing devices whereas Group 2 was tested on the newly designed device: the resistance-free gliding, ball-mounted platform. Stiffness (N/mm), yield Load (N), and failure Load (N) were registered. In the evaluation of both testing procedures, the results of the right and left tibiae were compared according to the rat they originated from.

**Results:**

Stiffness (S) showed highly significant differences (p = 0.002) with 202.25 ± 27.010 N/mm SD (Group 1) and 184.66 ± 35.875 N/mm SD (Group 2). Yield Load (yL) showed highly significant differences (p < 0.001) with 55.31 ± 13.074 N SD (Group1) and 37.17 ± 12.464 N SD (Group2). The mean failure Load (fL) did not differ significantly (p < 0.231) between Group 1: 81.34 ± 11.972 N SD and Group 2: 79.63 ± 10.345 N SD.

**Conclusions:**

We therefore conclude that, used in the three-point bending/breaking test, the mobile, ball-mounted platform device is able to efficiently eliminate the influence of friction in terms of stiffness and yield load. Failure Load was not affected. We suggest that the new ball-mounted platform device, when compared to other existing techniques, generates more accurate test results when used in the three-point bending/breaking test of the metaphysis of long bones.

## Background

Biomechanical testing of the structural properties of different skeletal phenotypes is an essential part of basic bone research. Tensile strength tests, bone compression tests, nano- and microindentation testing, torsional strength testing or three- and four-point bending/breaking tests are just a few examples of the biomechanical test variety available [1]. For the quantitative evaluation of the quality of long bones like the tibia, the most suitable and established method is the three-point bending and breaking assessment. In recent literature however, one drawback of this method has appeared. And this drawback we wanted to address in our study:

When a force is applied to the tibia metaphysis via the three-point bending/breaking test, the diaphysis lengthens along its axis, creating friction during this movement at the point of contact between the distal diaphysis and the solid aluminum block [2]. This frictional force could incorrectly increase the force needed to break the metaphysis.

Quantitative evaluation of bone becomes necessary especially when it comes to bone disorders. One of the largest concerns in modern society regarding bone disorders lies in the field of osteoporosis. The hallmark of osteoporosis is the deterioration of the trabecular bone structure possibly accompanied by a decrease in bone mineral density [3,4]. This deterioration occurs mainly in the trabecular bone architecture of the bone metaphysis, since trabecular bone is mainly found in the metaphyseal areas of long bones [5]. Further, osteoporotic bone fractures in locations with high concentrations of trabecular bone, such as the proximal and distal femur, the proximal tibia, the distal radius, and the vertebral bodies.

Recently there has been an increased research focus on this bone disorder because it affects a steadily increasing number of people [6-13]. The World Health Organization (WHO) has identified osteoporosis as one of the major health issues worldwide next to other major non-communicable diseases such as cardiovascular diseases, cancer, chronic respiratory diseases, and diabetes [14,15].

Consequently, health service costs are increasing. In the US, the cost of osteoporosis for the year of 2005 was estimated to range from $13.7 billion to $20.3 billion [16]. Further, the expenditures are expected to rise to $25.3 billion per year by 2025 [17]. Therefore, the importance of accurate biomechanical testing of trabecular bone has to be emphasized. In order to biomechanically evaluate osteoporotic bone, one has to strongly consider that this disease mainly affects the trabecular bone and thus the metaphysis [18-21].

The aim of this study was to address the fact that friction is a problem and alters the resulting data achieved in current standard three-point bending/breaking tests conducted on a solid aluminum block. Also, this paper aims to provide a technological solution for the elimination of this methodological problem by using a newly-designed mobile, ball-mounted platform that allows a friction-free lengthening of the diaphysis during the force application phase. Thus, only the force concerning solely the metaphysis, where trabecular bone is mainly located, is registered, providing a more accurate testing outcome. With this paper we want to facilitate and advance osteoporosis research in the future.

## Methods

### Ethical Approval and ARRIVE Guidelines

The study design was approved by the ethical committee of the German Landesuntersuchungsamt Koblenz, Rheinland-Pfalz and complied with all their animal research guidelines (animal research ethical approval number 23177-07/G12-7-027). Further, the animal research conducted in this study adheres to the ARRIVE guidelines as outlined by the National Centre of the Replacement Refinement and Reduction of animals in Research.

### Specimen preparation

70 tibiae form 35 three-month old female Sprague–Dawley rats were harvested. The fibulae were proximally detached from the tibia and distally removed at the synostosis. The prepared, tissue-free tibiae were then frozen in separate tubes without any medium or additional material inside the tubes by -20°C until use according to the established methodological approach [2,18,22,23]. We did not store them in saline soaked gauze or other liquid medium, because mechanical forces released through freezing liquid might damage the bone architecture. Freezing bone by -20°C for less than one year does not affect trabecular structure [24]. The tibiae were thawed wrapped in gauze moist with saline solution immediately prior to testing. The thawing-time for each tibia was kept constant. During the testing procedure itself the tibiae were removed from the moist gauze and not additionally moistened since the testing time always lasted less than 60 seconds. Both the aluminum block and the mobile, ball-mounted platform remained dry at all times. The tibiae were tested on two consecutive days, always completely testing one group on the same day to keep testing conditions as constant as possible, and thus avoiding potential data errors due to setup changes and altered testing conditions from aluminum block to mobile, ball-mounted platform.

### The three-point bending/breaking test

For animals larger in size, such as sheep, pigs, and dogs, four-point bending/breaking tests are the standard for testing trabecular properties [25-27]. For rats, where the bones are significantly smaller, the established and standard method of testing metaphyseal bone is the three-point bending/breaking test [2].

### The three-point bending/breaking device

**Figure 1:**
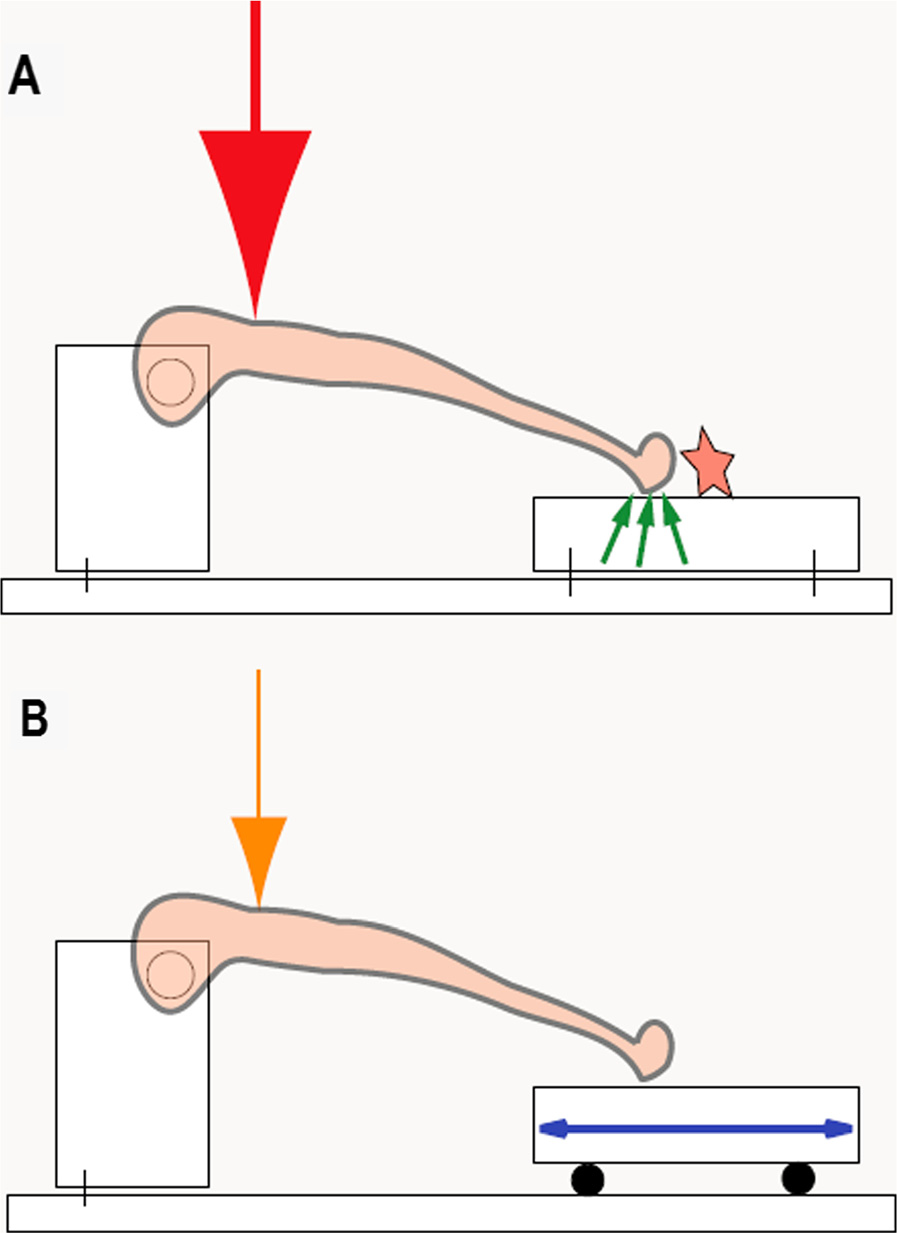
**Concept of the interaction of frictional forces and strength application needed for bending/breaking the tibia metaphysis.** Both constructions are designed to provide surface contact for the tibia at the same height. **A)** Frictional forces are created (small green arrows) when, through the axial lengthening of the tibia diaphysis, the distal tibia moves across the metal plate (star) and thus a larger force for bending/breaking is needed (large red arrow). **B)** Frictional forces are eliminated through the mobile, ball-mounted platform (blue double arrow) and less force is needed (orange arrow) to bend/break the tibia metaphysis.

**Figure 2:**
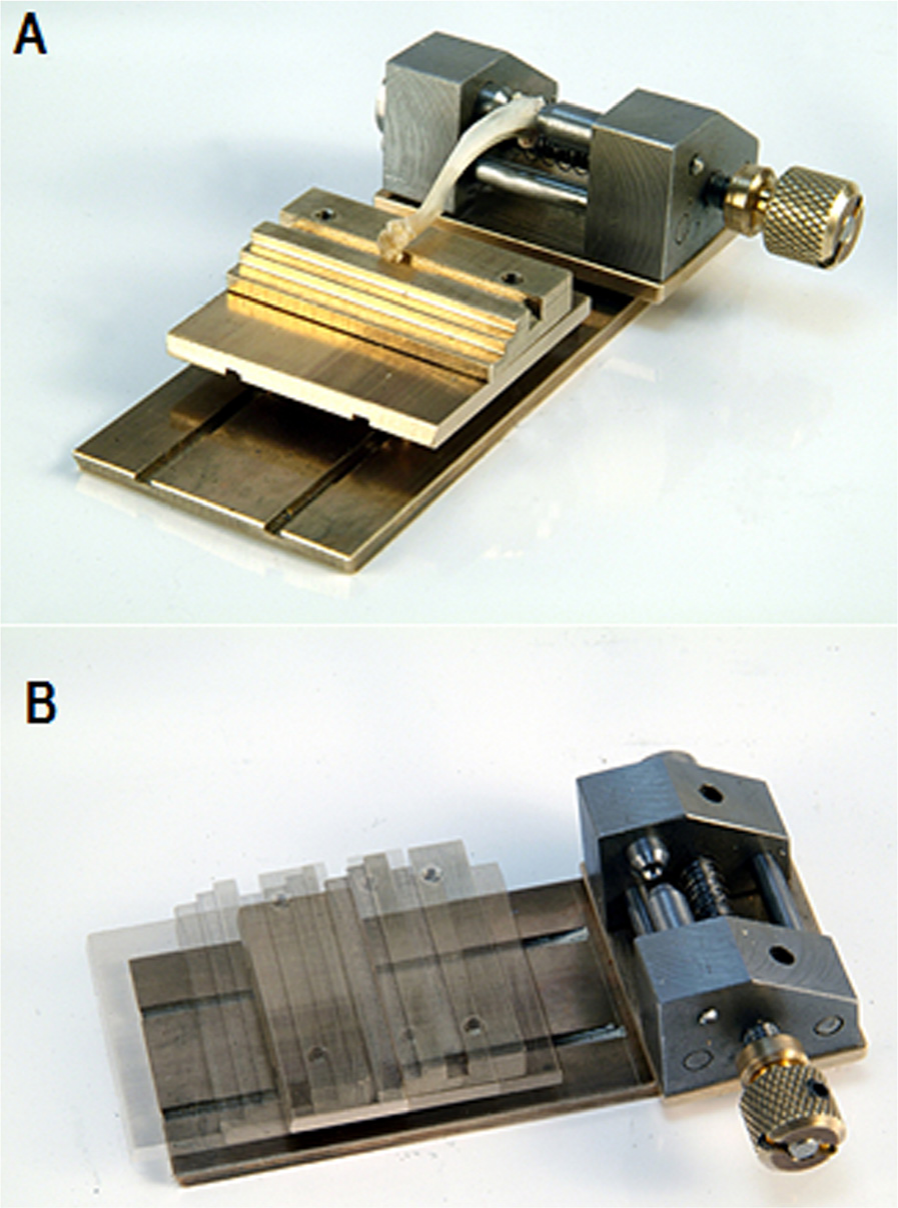
**The newly designed, mobile, ball-mounted platform for the three-point bending/breaking test. A)** The three-point bending/breaking device with the mobile, ball-mounted platform as a contact point for the distal diaphyseal tibia. **B)** Range of frictionless motion of the mobile, ball-mounted platform on the three-point bending/breaking device.

In order to eliminate unwanted frictional forces that occur during the lengthening of the tibia diaphysis in the previously used metaphyseal bending and breaking test designs, we created a new platform design for resistance free positioning of the bone (Figure 1).We consider the movement of the distal end of the tibia caused by the diaphyseal lengthening on a fixed surface as the essential problem, since through this movement unwanted friction is created. This friction inaccurately increases the necessary force that causes a bending or breaking in the metaphyseal tissue. The new mobile, ball-mounted platform is hypothesized to allow the tibia to lengthen completely free of resistance along its diaphyseal axis during testing and thus prevents development of unwanted friction caused by the movement of the distal diaphysis over the fixed aluminum block (Figure 2).Our three-point bending/breaking device for the rat (Figure 2) consisted of a base measuring 9.5 cm × 4 cm × 0.3 cm (length × width × height). On the upper side it had two 6.5 cm grooves which were 0.16 cm apart. Each groove was 0.01 cm deep and 0.02 cm wide and in each glided 5 stainless steel balls, with every ball having had a diameter of 0.04 cm.

The proximal end of the tibia was screwed in between two concave pins which were located opposite from each other in the head block with the concave ends facing each other. The pins were positioned through manual adjustment of a knob on the side of the head block. This secured both condyles and assured a customized fit for every tibia regardless of shape or size. Unlike in former trials [2], the proximal tibia epiphysis did not have to be removed. Dislocation of the proximal growth plate did not occur due to the even distribution of pressure on the pins.

The ball-mounted platform dimensions were 3.8 cm × 4 cm × 0.9 cm (length × width × height). It was made of aluminum and possessed an area of 1.4 cm × 4 cm (length × width) with a groove that was 0.03 cm wide and 0.01 cm deep. The distal end of the tibia rested in this groove during testing. The height of the mobile, ball-mounted platform, when placed atop of the 10 balls, was 1.3 cm, which equaled the same height as to when the aluminum block was used. On the bottom of the ball-mounted platform, opposite the upper side of the base, there were two grooves which had the same location and dimensions as the two groves on the base described earlier.

**Figure 3:**
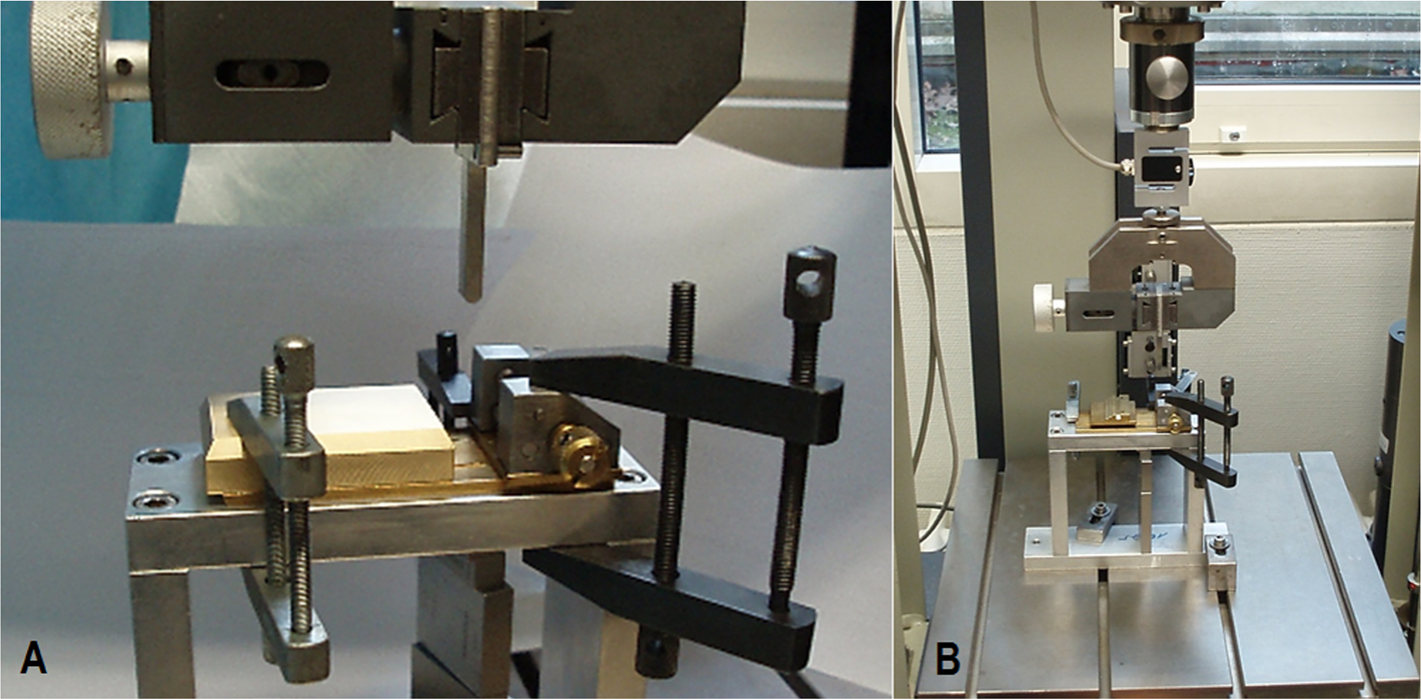
**Three-point bending/breaking test set-up with the two different platform options. A)** The three-point bending/breaking device strapped into a ZWICK-testing machine type Z020/TND (ZWICK-/Roell, Ulm, Germany) with a solid aluminum block as contact point for the distal diaphyseal tibia. **B)** Overview of the ZWICK-testing machine and the three-point bending/breaking device with the mobile, ball-mounted platform as contact point for the distal diaphyseal tibia.

“Plastilube” (Henkel AG, Duesseldorf, Germany) water-resistant ball-bearing fat was used to keep the steel balls moving smoothly and virtually frictionless in their designated grooves.The block used for the testing was 6 cm × 4 cm × 1.3 cm. It was made of aluminum and was polished to display a smooth surface (Figure 3A).

### Mechanical testing machine

For the mechanical testing, we used the ZWICK-testing machine type Z020/TND (ZWICK-/Roell, Ulm, Germany) (Figure 3B). The machine had a measuring range from 0.4 - 200 N at a relative accuracy, starting at 1.0 N, of +/- 0.06%. For recording of the force application on the tibia, the software “testXpert” was used. The speed of the feed-motion was set to 1 cm/min. The trial was ended automatically when the linear displacement distance of the strength application stamp exceeded 0.4 cm or if the strength dropped more than 80% of the maximum strength applied.

### Mechanical testing procedure

Tibiae were split into two groups based on right or left side. The group with the right tibiae had the three-point bending/breaking test administered with the distal diaphysis on the solid aluminum block: Group 1. The group with the left tibiae had its three-point bending/breaking test administered with the distal diaphysis on the new ball-mounted, mobile platform: Group 2.

**Figure 4:**
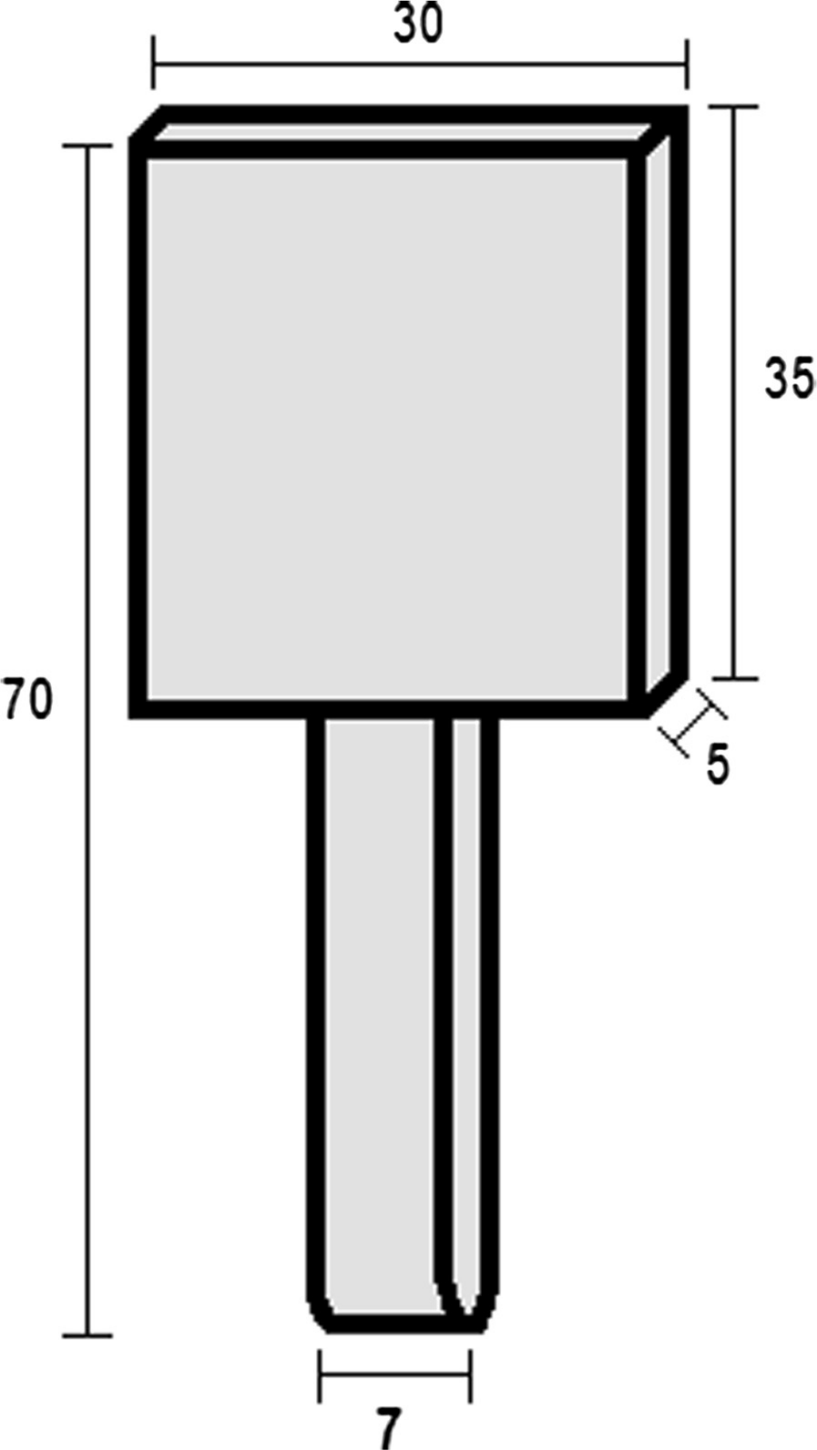
**Schematic drawing of the stamp which was connected to the ZWICK-testing machine and lowered onto the marked location of tibia metaphysis.** The radius of the tip is 2.5 mm. The dimensions in the drawing are all in millimeter (mm).

A distance of 5 mm distal the epiphyseal line was measured and marked to define the metaphysis. Then the condyles of the proximal tibia were placed into the head block in between the two pins and fitted through manual adjustment. The distal diaphysis was placed, depending on which group the tibia belonged to, either on the surface of the aluminum block or into the 0.03 cm wide and 0.01 cm deep groove of the ball-mounted, mobile platform.The stamp, which was connected to the ZWICK-testing machine type Z020/TND (ZWICK-/Roell, Ulm, Germany), was 3.45 cm long, 0.7 cm wide and 0.25 cm in diameter with a rounded tip (Figure 4). The stamp was aligned in such a manner, that it would administer the pressure directly onto the marked metaphyseal area, measured 0.5 cm distal of the epiphyseal line. It was automatically lowered onto the ventral metaphysis of the tibia to start the three-point bending/breaking test.

### Statistics and evaluation

**Figure 5:**
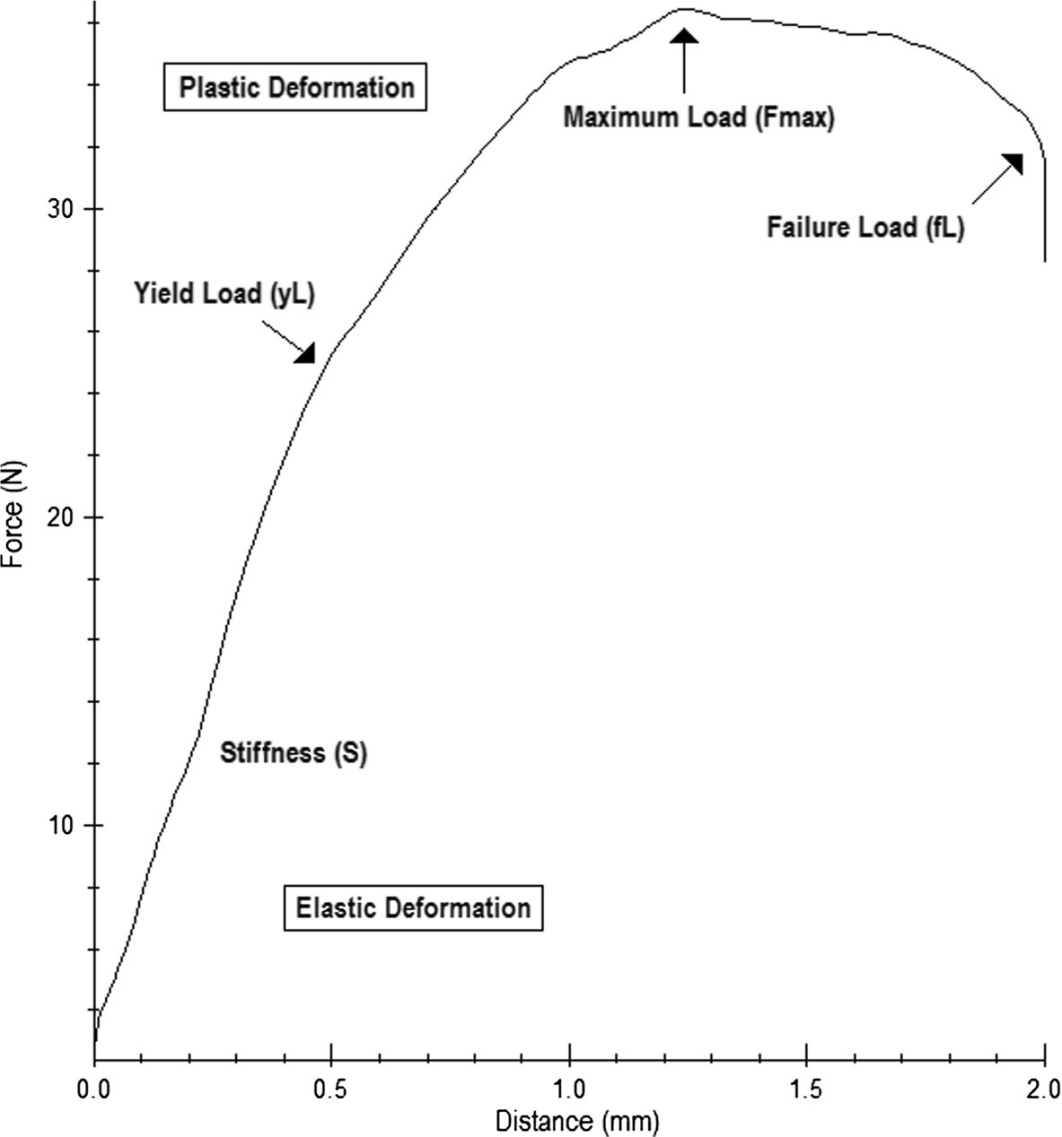
Example of the graphical visualization of the data (distance (mm) travelled by the stamp and force (N) exerted by the stamp) recorded by the “testXpert” software during the bending and breaking test performed on the newly designed mobile, ball-mounted platform.

During the trials, the “testXpert” software continuously recorded the force (in newtons) which was applied via the stamp on the tibia metaphysis. The force (in newtons) was graphically plotted against the stamp’s traveled distance (Figure 5). From this graph, the stiffness (S), which is defined as the resistance an elastic body exerts against deformation [28], was represented by the slope of the curve prior to the yield load. The yield load (yL) is the point where elastic deformation transforms into plastic deformation and first microfractures occur. It was assessed as a decrease in stiffness of more than twice the SD. The highest point on the graph, where the largest force was applied onto the tibia, was assessed as the maximum Force (Fmax). We also calculated the failure Load (fL) which is the force (in newtons) at the point of breakage.

The statistical evaluation of the different values for stiffness, yield load, and failure load for the tibiae tested on the aluminum block and the tibiae tested on the mobile, ball-mounted platform were analyzed in a paired t-test where p < 0.005 was considered to be significant. In the evaluation of both testing procedures, the results of the right and left tibiae were compared according to the rat they originated from.

## Results

**Figure 6:**
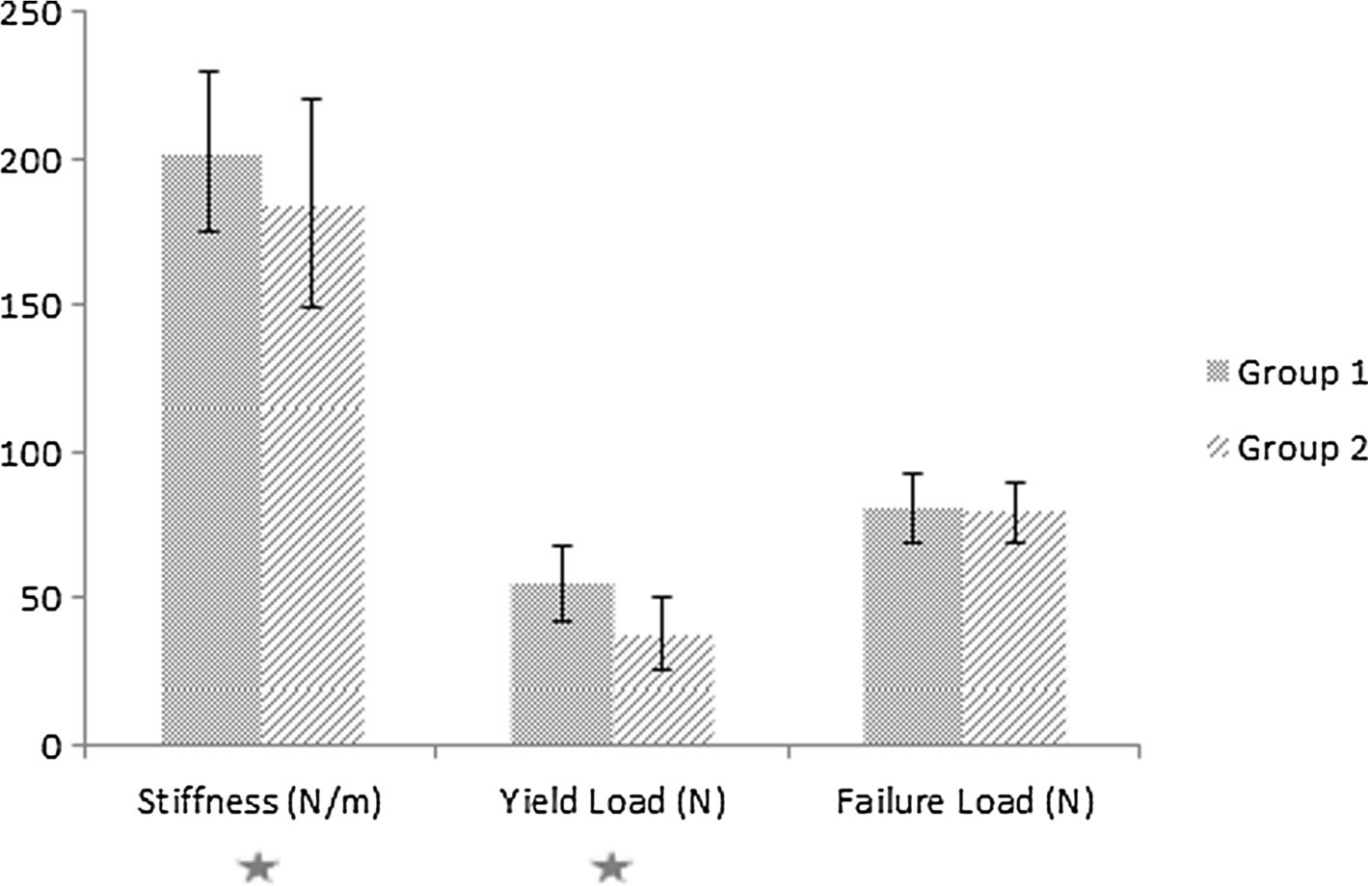
**Results for stiffness, yield load, and failure load of the three-point bending/breaking test for Group 1 (solid aluminum block) and Group 2 (mobile, ball-mounted platform).** The differences for stiffness were significant (p < 0.002) and the differences for yield load were significant (p < 0.000) (stars).

The mean stiffness for Group 1 was 202.25 N/mm ± 27.010 N/mm SD. The mean stiffness for Group 2 was 184.66 N/mm ± 35.875 N/mm SD. The results of the stiffness were significantly different (p = 0.002). The mean yield Load for Group 1 was 55.31 N ± 13.074 N SD. The mean yield Load for Group 2 was 37.17 N ± 12.646 N SD. The results were highly significant (p < 0.001). The mean failure Load for Group 1 was 81.34 N ± 11.972 N SD. The mean failure Load for Group 2 was 79.63 N ± 10.345 N SD. The differences were not significant (p < 0.231) (Figure 6).

## Discussion

Bone tissue research is becoming a progressively more interesting field of research for multiple medical specialties such as orthopedic-, trauma- and plastic surgery, but also for osteology and endocrinology due to the various changes in bone homeostasis caused by systemic diseases. An elemental part of all these research studies is the assessment of the quantitative properties of bone, which are extensively evaluated by biomechanical testing technologies.

Long bones consist of three main parts: the diaphysis, consisting mainly of cortical bone, the metaphysis, containing mainly cancellous bone, and the epiphysis. Cancellous bone is extremely sensible to changes in bone-mineral homeostasis and is therefore the area first affected by developing osteoporosis. To biomechanically evaluate these areas, special aspects and requirements have to be considered. First, a stable fixation of the bone has to be assured to avoid sudden changes in position while the stamp drives down during force application. To address this aspect, various possibilities have been designed. In previous studies, one approach was to fix the rat bone, a rat femur in these cases, but we consider it nonetheless just as relevant for the rat tibia, on a metal block with a proximal deepening in which the proximal bone could be placed during testing [6,7,29]. The shaft was located between to cylinders capable of rotation [6,7,29]. The distal end was placed onto the plane surface of the block [6,7,29].

Secondly, the shape and dimensions of the stamp are important. It should not apply the force only to a point shaped area, but in a transversal manner across the metaphyseal area in order to apply the force in an evenly distributed fashion. We therefore used the above mentioned design (Figure 4). Thirdly, it has to be made sure that during testing only the metaphyseal part is analyzed, and that the results are not influenced by the diaphyseal part of the bone. This influence of the diaphyseal part is the critical aspect which could, until now, not be eliminated via the existing testing devices. The distal part of the tibia moves over a fixed metal block during diaphyseal lengthening [2,18,30,31]. By moving in this described manner, we propose that the distal diaphysis was transferring the arising frictional forces created by this movement of bone over the aluminum block to the stiffness and yield load properties of the metaphyseal area.

In order to eliminate this falsification caused by the arising frictional forces during testing, there is a need to position the distal diaphysis on a surface that allows a resistance-free gliding during testing.

To achieve this, we decided on a mobile, ball-mounted technique.

During biomechanical testing, a lengthening of the rat diaphysis occurs of about 0.2-0.3 cm. In order to ensure a stable gliding of the mobile, ball-mounted platform, measuring 3.8 cm × 4 cm × 0.9 cm (length × width × height) with a top area of 1.4 cm × 4 cm (length × width), we used a consecutive series of 5 stainless steel balls on each side with a distance of 0.5 cm between the balls. To fully eliminate friction, a special silicone based ball bearing fat called “Plastilube” (Henkel AG, Duesseldorf, Germany) had to be applied to the balls.

For our study, we regarded the recently used techniques [2,18,30,31] as state of the art, and respectively as a negative control. Thus, the testing of Group1 was performed according to their described methodologies, in which the distal tibia diaphysis is positioned on a fixed aluminum block [2,18,30,31].

The differences of our results between the two plate designs are apparent. The stiffness of group 1 was around 20 percent higher than the stiffness of Group 2. Since the term stiffness describes the resistance of a tissue against an incoming force [28], these results demonstrate the influence of the frictional forces arising from the movement of the distal diaphysis over a fixed metal plate on the metaphyseal biomechanical properties. Thus, the measured results for the stiffness are falsified by these frictional forces. In terms of the yield load, this falsification is even more evident. Here the change from elastic to plastic deformation under the applied force is significantly enhanced in Group 1. Interestingly, the results found in the final part of the testing procedure, the failure load, were very similar. Thought should also be given to the applicability of these findings in regard to larger animal models as well as further examinations of cancellous bone of the metaphysis of, for example, the humerus, the femoral neck or the distal radius.

Further we do not want to hesitate to mention the limitations of this study. One limitation would be that, even though using bones from the same animal in comparison is a generally accepted method (since the conditions such as nourishment, exercise, mechanical strain, environmental conditions etc. are as much the same as possible for the animal and thus for the bones), the procedure of comparing the bones of the same animal to one another do not have an independent confirmation. Therefore it cannot be proven that the tibiae of the same rat have a comparable bone architecture and strength. For similarity of the bones to be not just highly possible but proven, there should be, in future experiments, an investigation via micro CT, BMD, or a measurement of trabecular bone quantity.

Other limitations of this study could be improved with a micro-camera. We did not utilize one because this was a project purposed to identify if there was at all an influence and thus a difference in the two setups concerning friction during the three-point bending/breaking test. Now, considering the presented results, we recommend the utilization of a micro-camera in future follow-up experiments to go into more detail on the influence of friction on the outcome of the three-point bending/breaking test. For example, had we had data from a micro-camera, it would have been possible to record the exact distance of the diaphyseal lengthening on the aluminum block and so to produce force-displacement curves of this matter. Also, we could have numerically assessed in which phase of loading most of the diaphyseal lengthening occurred. Macroscopically most of it occurred in the early phase of loading, which we attribute to the elasticity the bone displays, before it, under further loading, transits into the phase of plastic deformation. Another limitation concerning the lack of a micro-camera would be the fact that with one, it might be possible to calculate such values as the frictional coefficient, the frictional force itself, and the contribution of friction to the experiment. Therefore, again, we suggest this to be included in follow up studies.

It would be interesting, since the animals used in this study did not suffer from the condition of osteoporosis and since osteoporosis is the most common disease which requires testing of the metaphyseal area of long bones, to examine osteoporotic bone with both presented testing devices. Unfortunately this suggested approach was not covered by our animal protection committee in the case of this study. However, we would like to potentially follow up on this matter.

## Conclusions

In conclusion, the newly designed mobile, ball-mounted platform device for biomechanical testing was used to evaluate the biomechanical properties of the metaphysis of long bones, in this case of rat tibiae. Through eliminating the influence of friction of the previously used device-design, which used a solid aluminum block, this new, ball-mounted platform device produced more real and accurate results for the biomechanical properties of the tibia metaphysis. Although the new device is only a small stone in the mosaic that is the whole biomechanical testing process, we may recommend it for further use of all metaphyseal biomechanical testing endeavors to achieve accurate and realistic data.

## Abbreviations

SD: Standard deviation; S: Stiffness; yL: Yield load; fL: Failure load; N: Newton; Mm: Millimeter; Cm: Centimeter; Fmax: Maximum force.

## Competing interests

There are no competing interests, neither financial nor non-financial.

## Authors’ contributions

GAM was responsible for the validation of the new device, conduction of the testing, and writing of the paper, since she is a native speaker. LH is the corresponding author and leader of the project. She was responsible for the supervision of the project and she was involved in the technical development of the machine. She also participated in manuscript preparation. CH participated in animal preparation and manuscript preparation. DK participated in animal preparation and manuscript preparation. HH was substantially responsible for the machine development. BH did the statistical analysis of the data. UK gave substantial input concerning the study design and participated in manuscript preparation. All authors read and approved the final manuscript.

## Authors’ information

GAM was the co-PI (co-principal investigator).

## Pre-publication history

The pre-publication history for this paper can be accessed here:

http://www.biomedcentral.com/1471-2474/15/245/prepub
